# Artificial Urinary Sphincter With Bilateral Atrophic Kidneys and Accessory Renal Arteries in a Male Cadaveric Subject: A Case Report and Clinicopathological Reconciliation of Urinary Abnormalities and Embryogenetic Correlation of Vascular Aberrations

**DOI:** 10.7759/cureus.37948

**Published:** 2023-04-21

**Authors:** Sanjoy Sanyal, Gomattie Chunilall, Ginikachukwu O Uzoekwe, Edward Peter Taylor

**Affiliations:** 1 Surgical Anatomy, Richmond Gabriel University College of Medicine, Belair, VCT; 2 Academic Affairs, Richmond Gabriel University, Belair, VCT; 3 Medical Education and Simulation, Richmond Gabriel University College of Medicine, Belair, VCT; 4 Medicine, Richmond Gabriel University, Belair, VCT; 5 General Surgery, Maypen General Hospital, Clarendon, JAM; 6 Internal Medicine, Richmond Gabriel University College of Medicine, Belair, VCT

**Keywords:** physician guideline adherence, hagen-poiseuille law, embryogenetic correlation, cadaver dissection, clinicopathological correlation, urinary incontinence, accessory renal arteries, bilateral renal atrophy, artificial urinary sphincter

## Abstract

A unique combination of triple abnormality in a willed male body donor dissection, with putative clinicopathological correlations during the subject's lifetime, is described in this case report. The subject had a three-piece artificial urinary sphincter surgically implanted around the proximal corpus spongiosum, left scrotal pouch and in the lower left abdominal wall, ostensibly for urinary incontinence during his lifetime, though the etiology of the latter was not immediately obvious. He also had a total of three accessory renal arteries involving both sides, complicated by bilateral diffuse renal atrophy from presumable glomerulosclerosis or nephrosclerosis-induced nephrotic syndrome. While each entity may not be so unique per se, each is not too common either. The combination of all three findings has not been described to date in the contemporary literature in a single male cadaver dissection. Only seven reports of artificial urinary sphincter studies on human cadaver subjects could be detected in contemporary literature, this being the eighth. Finally, there were no apparent etiopathological or pathogenetic mechanisms to explain the occurrence of each or the coexistence of all of them in a single male cadaveric subject. The artificial urinary sphincter was reviewed with respect to its characteristics, placement, and efficacy. An attempt was made to establish the cause-effect relationship between the artificial sphincter and urinary incontinence that necessitated the implant. Thereafter, a clinicopathological correlation was proposed in this case report to reconcile the concomitance of urinary incontinence, bilateral accessory renal arteries, and bilateral renal atrophy. An embryogenetic mechanism of the aberrant renal arteries was also suggested. Physician awareness from the standpoint of preoperative investigation of such cases was also highlighted.

## Introduction

Ever since the first artificial urinary sphincter (AUS) surgery was performed in 1972[1], AUS implants, techniques of their insertion, and ease of control for the patient have made exponential advancements over the last few decades [2]. Recently during abdominal and perineal dissection in the cadaver lab of our university medical college for surgical teaching purposes, we incidentally detected and explanted a partially functioning three-piece AUS implant in toto from a male cadaveric subject. It was inserted during his lifetime, ostensibly for urinary incontinence (UI). The most common cause of male UI is benign prostatic hypertrophy (BPH), prostatectomy for BPH or cancer, and prostatic irradiation for cancer [2,3]. However, in our subject, there was no objective evidence of any of these.

Extension of our dissection into the retroperitoneal region also incidentally revealed three long, thin, accessory renal arteries (ARA) in total involving both sides, in addition to the main renal arteries (MRA) of normal caliber in their respective hila. Systematic reviews and meta-analyses of ARA literature and several smaller studies have established that a single ARA in an individual, as diagnosed incidentally by computerized tomogram (CT) or magnetic resonance imaging (MRI), is not uncommon. The prevalence ranges from 20-30% of the population. However, multiple ARAs in a single individual have only 0.1*-*2% of prevalence [4-6].

ARA, with or without stenosis, may be accompanied by hypertension (HTN) during the lifetime of the subject, but not necessarily so [7]. Though none of the arteries in our subject, aberrant or regular, were stenotic, the subject had bilateral diffuse renal atrophy (RA) in the presence of normal ureters and urinary bladder. Atrophic kidneys can result from numerous systemic or urological illnesses. RA may or may not be associated with HTN during the lifetime of the subject and is not always accompanied by ARA [7,8]. As such, the coexistence of ARA and RA is very rare [9]. The uniqueness of this case report derives from the coexistence of three rather uncommon conditions, namely AUS implant, bilateral ARA, and bilateral RA, without apparent etiopathological correlation between them. Despite best efforts, we did not find any report of all three findings in a single adult male cadaveric subject.

The AUS implant was analyzed with respect to its genesis, placement, functioning, and efficacy by comparison with seven other cadaveric studies. Thereafter, correlating what was known about the subject during his lifetime, what was found in the dissection, and arriving at a clinicopathological reconciliation between bilateral ARA, bilateral RA, the AUS implant itself, and the presumed UI that necessitated it, was a major focus of this case report. The ARAs and RAs detected in our subject were reviewed in isolation and in combination. Proposing an embryogenetic basis for the ARAs in our subject was a secondary objective of this case report. Concluding physician guidelines from the standpoint of preoperative investigation of such cases were also proposed.

## Case presentation

A male cadaver wrapped in a sheet saturated with 37% formalin and 5% of 85% phenol disinfectant and sealed in a 6Mil (6/1000th inch) low-density polyethylene (LDPE) bag was procured from a licensed embalmer from New Jersey, USA after it was approved for release by the state government. The body had been embalmed with 10 gallons of a solution consisting of 25% of 85% phenol solution, 25% of diethylene glycol, 25% of water, and 25% of alcohol (1/3^rd ^methanol and 2/3^rd^ ethanol). To this mixed solution, three ounces of formalin were added to each three gallons of solution. The cadaver was a donated non-clinical human tissue on loan from US medical schools, with written permission for it to be exported for medical research and education purposes to our university medical college. It was incumbent on the borrowing school to ensure the dissected remains were imported back to the lending institution in the US for cremation under regulations stipulated in the Pan American Health Organization XVII Resolutions. A donor form giving permission from the executor of the deceased is on file at the lending institution in the US. This form may not be released to anyone because of Health Insurance Portability Accountability (HIPAA) Law, which was enacted in the US to give privacy to all patients' medical information.

As per legally disclosed information at the receiving institution, during the end of life, the subject was a 78-year-old obese African-American male who was certified as being free from all communicable diseases. The subject was college-educated, married, and had served in the armed forces. The immediate cause of death was coronary artery disease (CAD). Antecedent causes were supraventricular tachycardia (SVT) and severe obstructive sleep apnea (OSA).

**Figure 1:**
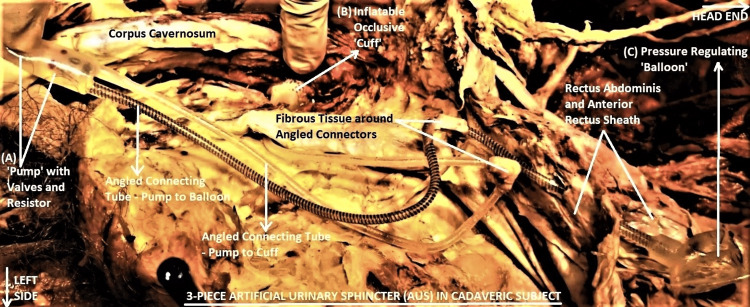
Dissected image showing a three-piece artificial urinary sphincter (AUS) in situ (A) "pump" in the left scrotal region, (B) "cuff" around the proximal part of corpus spongiosum (indicated by the pointing finger of a surgeon), (C) "balloon" under the left rectus sheath in the lower abdominal wall. Two angled connecting tubes, one of them with spiral black markings, are also labeled. The head-end of the subject is to the right of the picture frame, and the left side is on the lower part of the frame. All subsequent pictures have the same orientation.

The lower anterior abdominal wall and perineal dissections in the subject incidentally revealed a three-piece AUS implant composed of a silicone elastomer filled with a clear fluid. The three pieces were connected by two kink-resistant tubes: one with black spiral markings and the other clear, and they were joined by angled sutureless connectors. Especially the angled regions of the tubes were encased in a dense layer of fibrotic tissue. The left lower anterior abdominal wall and penoscrotal regions had dense postoperative fibrous tissue with remnants of blue polypropylene suture materials embedded inside (Figure 1).

**Figure 2:**
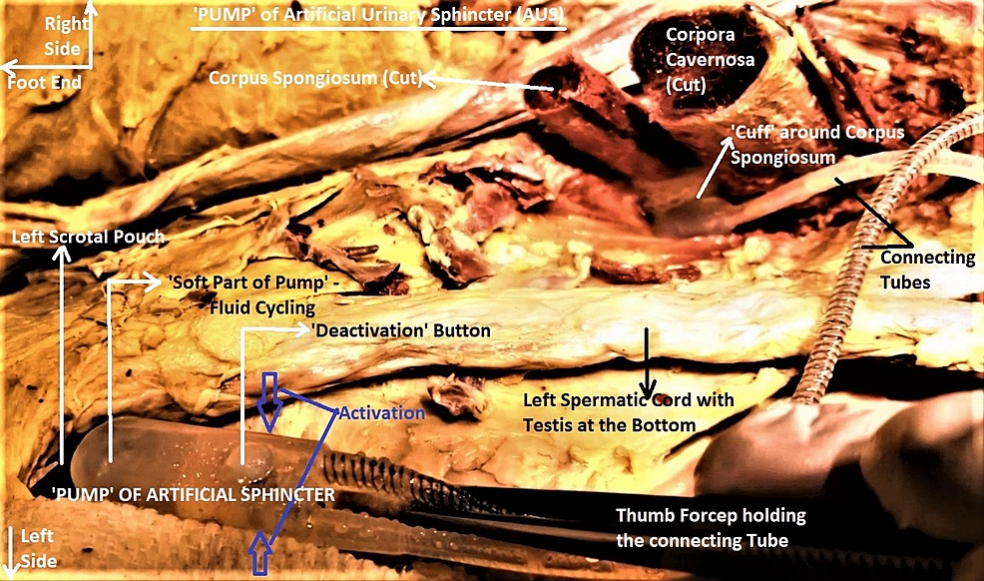
Dissected image enlarged to show the location of the pump of AUS in the left scrotal pouch. Note the pump proper in the lower part of the image, the deactivation button in the upper part, and the two tubes leading out from the pump. The site for activation is indicated by blue arrowheads. Part of the cuff is also seen around the proximal corpus spongiosum, which has been transected to permit visualization of the urethral cut end. The orientation of the subject was the same as in Figure 1. AUS -  artificial urinary sphincter

**Figure 3:**
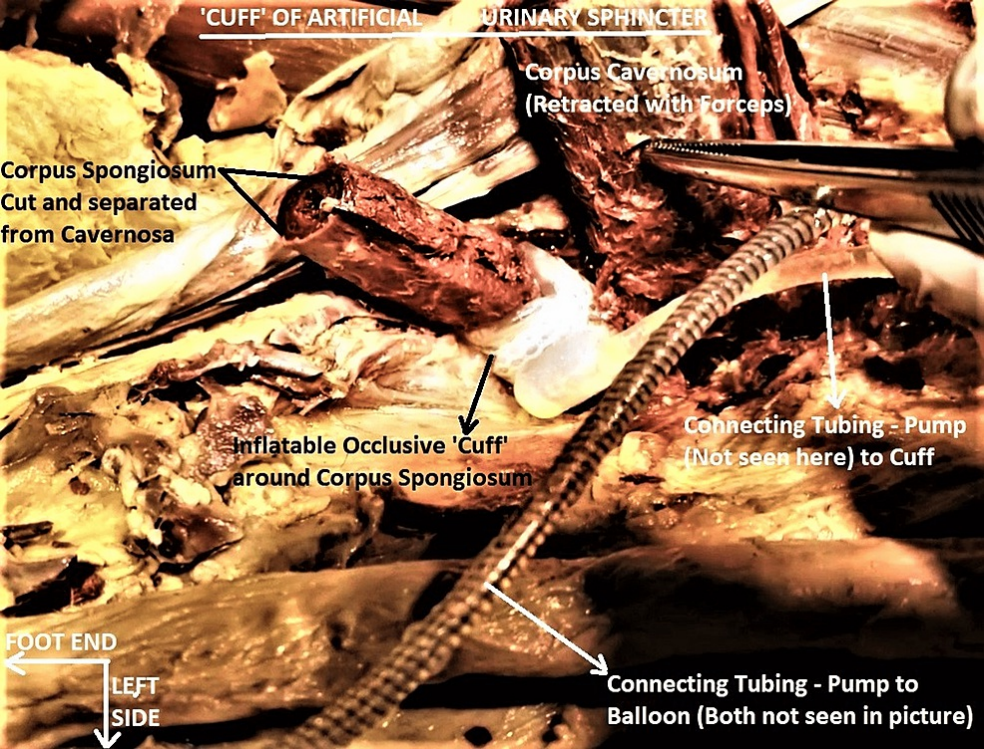
Dissected image enlarged to show the cuff of AUS around proximal corpus spongiosum The corpus spongiosum has been transected transversely and separated from corpora cavernosa for better visualization of the urethral cut end. There were no signs of pressure atrophy or urethral erosion from the AUS. Connecting tubing from the pump to the balloon, with black spiral radiopaque markings, is partly visible in the foreground. The orientation of the subject is the same as in previous pictures. AUS -  artificial urinary sphincter

The AUS implant consisted of a "pump", a "balloon", and a "cuff" (Figure 1). The pump was implanted in a left scrotal sub-dartos pouch. The lower half of this piece was the pump proper, while the upper half had a deactivation button on its surface and housed the inflow, outflow check valves, the deactivator valve poppet, and the hydraulic resistor (Figure 2). The balloon reservoir was implanted under the left lower anterior rectus sheath in a shallow pouch on the surface of the left rectus abdominis (RA) muscle, where it produced slight pressure atrophy of the muscle. The balloon was flaccid and partially empty. The cuff was double-layered, flat-backed, 2 cm wide, and circumferentially encircling the corpus spongiosum, just proximal to its confluence with the corpora cavernosa (Figure 3).

In vivo testing of AUS revealed the connecting tubes were patent, and all components were partially functional. Squeezing the pump enabled fluid to flow to and from the balloon and cuff, both of which alternately and reciprocally inflated or deflated. The nature of the clear fluid in the system could not be determined but was presumed to be saline mixed with radio-opaque contrast. There were no signs of inflammation of the tissues from the material of AUS. The corpus spongiosum did not show signs of pressure atrophy. The interior of the spongy urethra, as visualized through its distal cut end, did not reveal any evidence of urethral erosion (Figure 3). The urinary bladder was normal externally and internally, including the mucosa, trigone, detrusor, internal urethral orifice, and ureteric orifices. Both ureters were unremarkable throughout their length.

**Figure 4:**
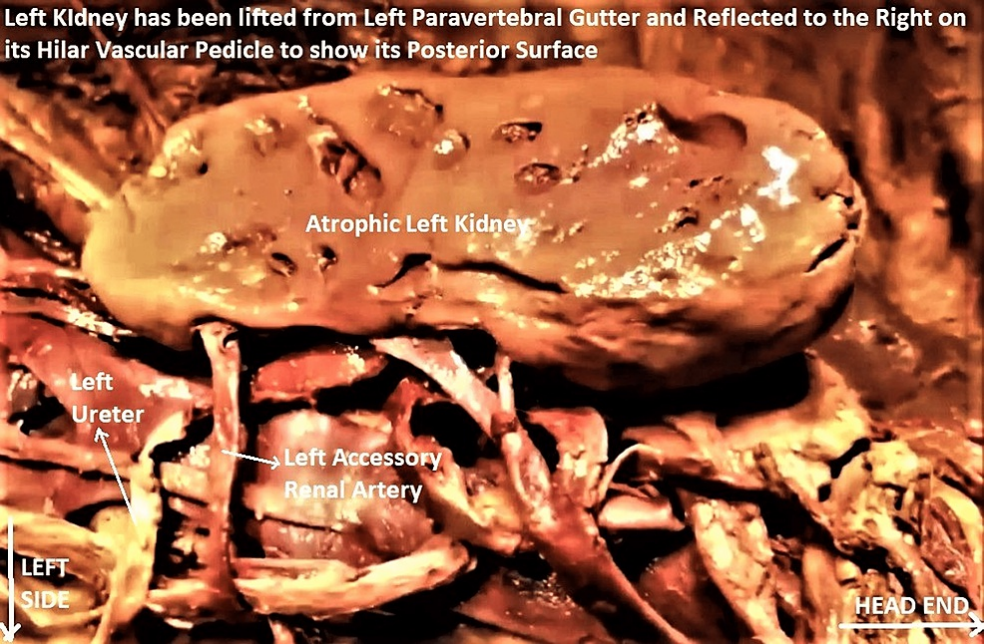
Enlarged image of abdominal dissection to show atrophic left kidney It has been eviscerated from the left paravertebral gutter of the subject and reflected to the right to show its posterior surface. Note the scarred, pitted, pock-marked posterior cortical surface. Incidentally, the left accessory renal artery (labeled with a white arrow) can be clearly seen entering the kidney separately through the lower polar cortex. It was running behind the ureter (also labeled with a white arrow). The orientation of the subject is the same as in previous images.

**Figure 5:**
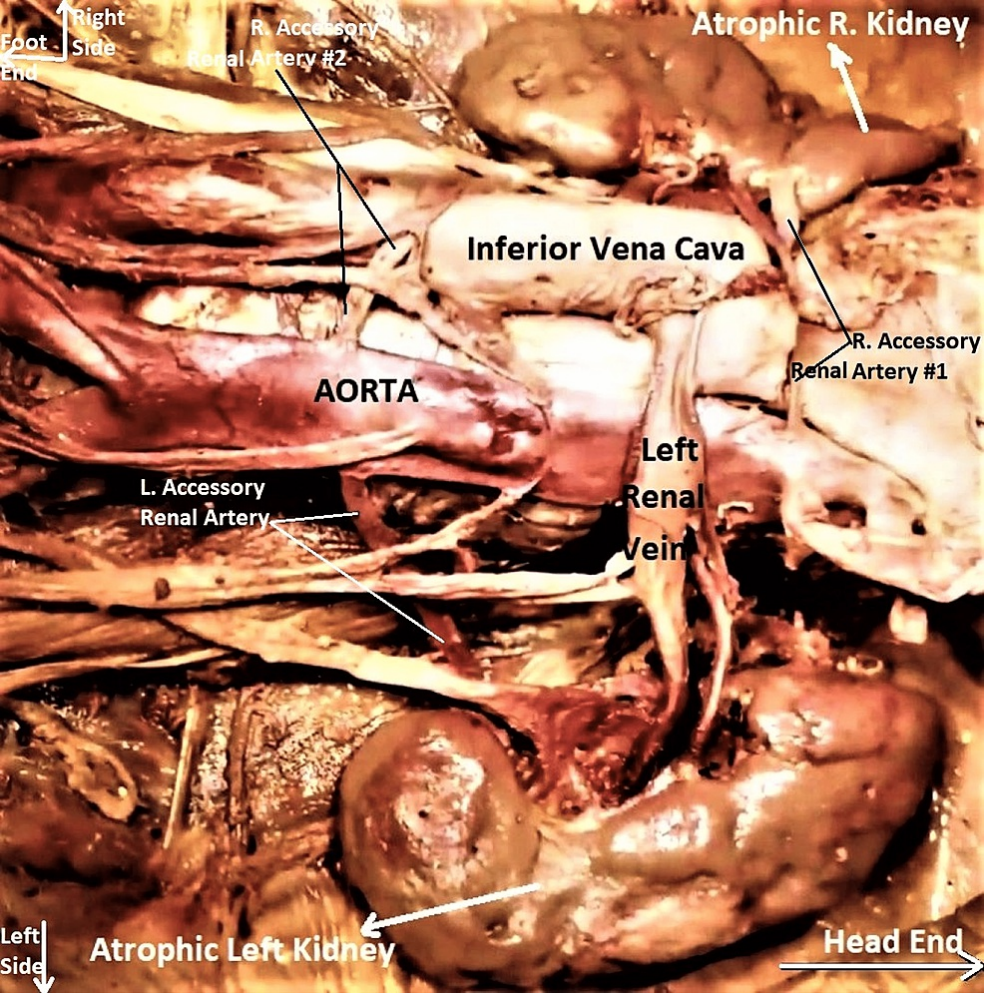
Enlarged image of abdominal dissection to show both atrophic kidneys in respective para-vertebral gutters Both kidneys are small and shrunken. Note the scarred, pitted, pock-marked cortical surfaces of both kidneys. Incidentally, this image also shows three accessory renal arteries, which are more clearly highlighted in Figures 9 and 10. The orientation of the subject is the same as in previous images.

**Figure 6:**
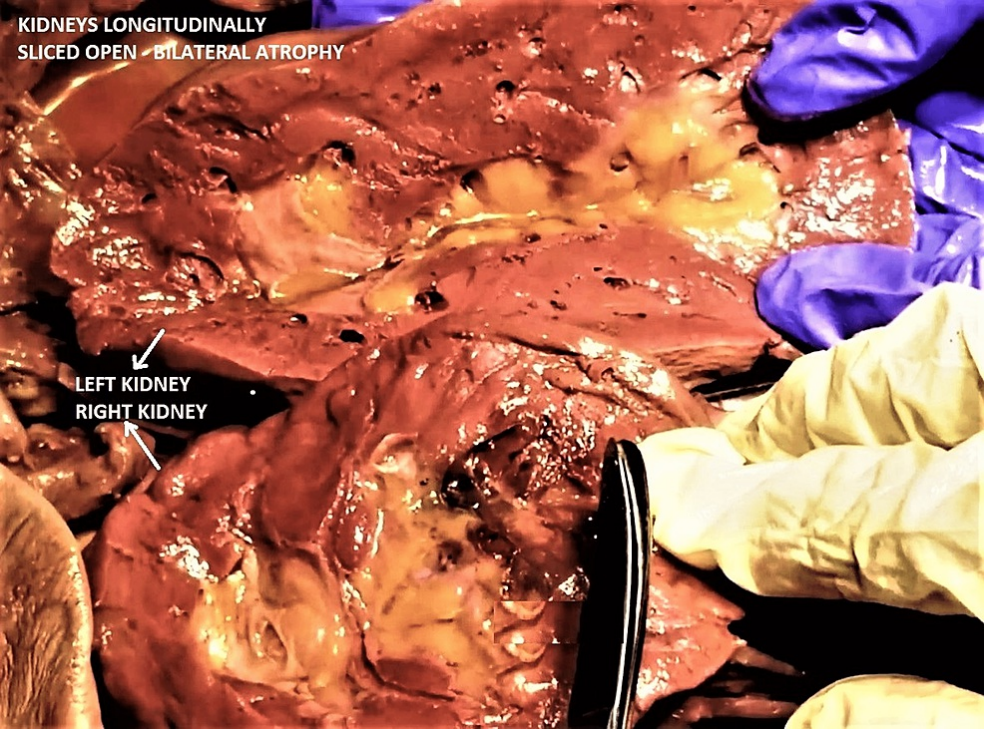
Enlarged image showing longitudinal cut surfaces of both kidneys, held alongside each other Note the thin atrophic cortex, absent cortico-medullary differentiation, with compensatory mild pelvicalyceal dilatation. The kidneys show a pitted appearance on cut sections. Compare with a normal kidney in Figure 7.

**Figure 7:**
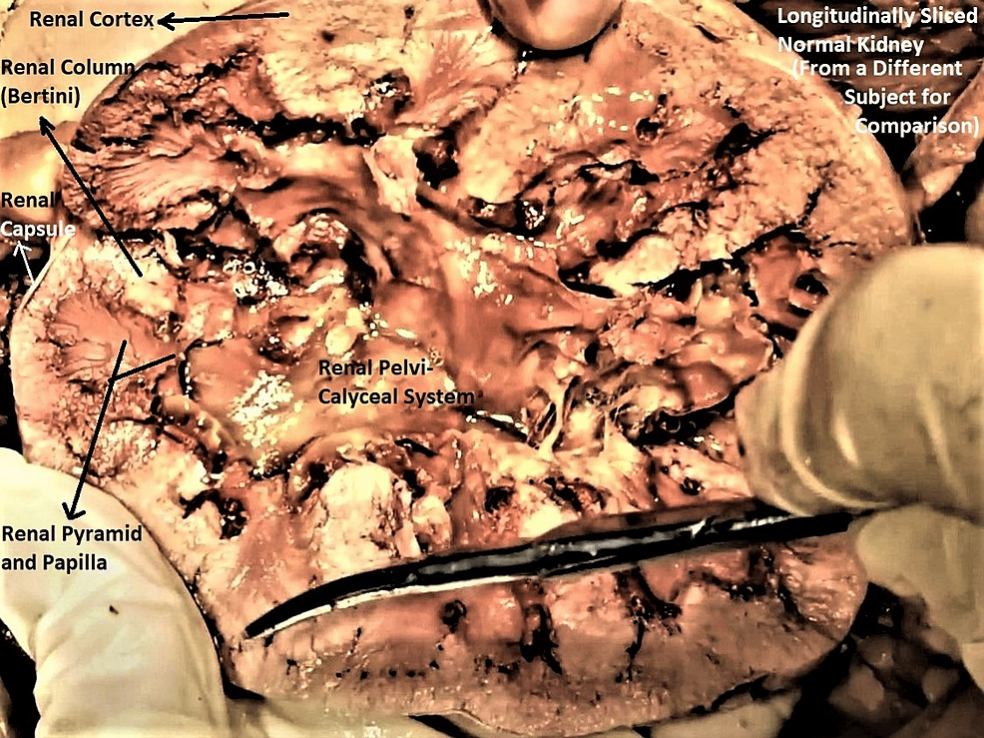
Image of a longitudinally sliced normal kidney from a different subject This is for comparison with the sliced kidney from the subject in our study (Figure 6). Note the clearly demarcated renal capsule, cortex, medulla, medullary pyramids, papilla, and normally configured pelvicalyceal system in this dissected specimen, all of which are labeled with arrows.

Both kidneys were small, soft, flabby, and diffusely atrophic. Both had scarred, pock-marked and pitted cortical surfaces with grooves and ridges. Longitudinal cut sections of both kidneys revealed diffuse bilateral renal cortical atrophy, poor cortico-medullary differentiation, the honeycombed appearance of renal parenchyma, and a mildly dilated pelvicalyceal system (Figures 4-6). The histological section of the subject's kidney was not available, though gross comparison with a cut-section of a normal kidney from a different cadaveric subject reinforced the abovementioned findings (Figure 7).

**Figure 8:**
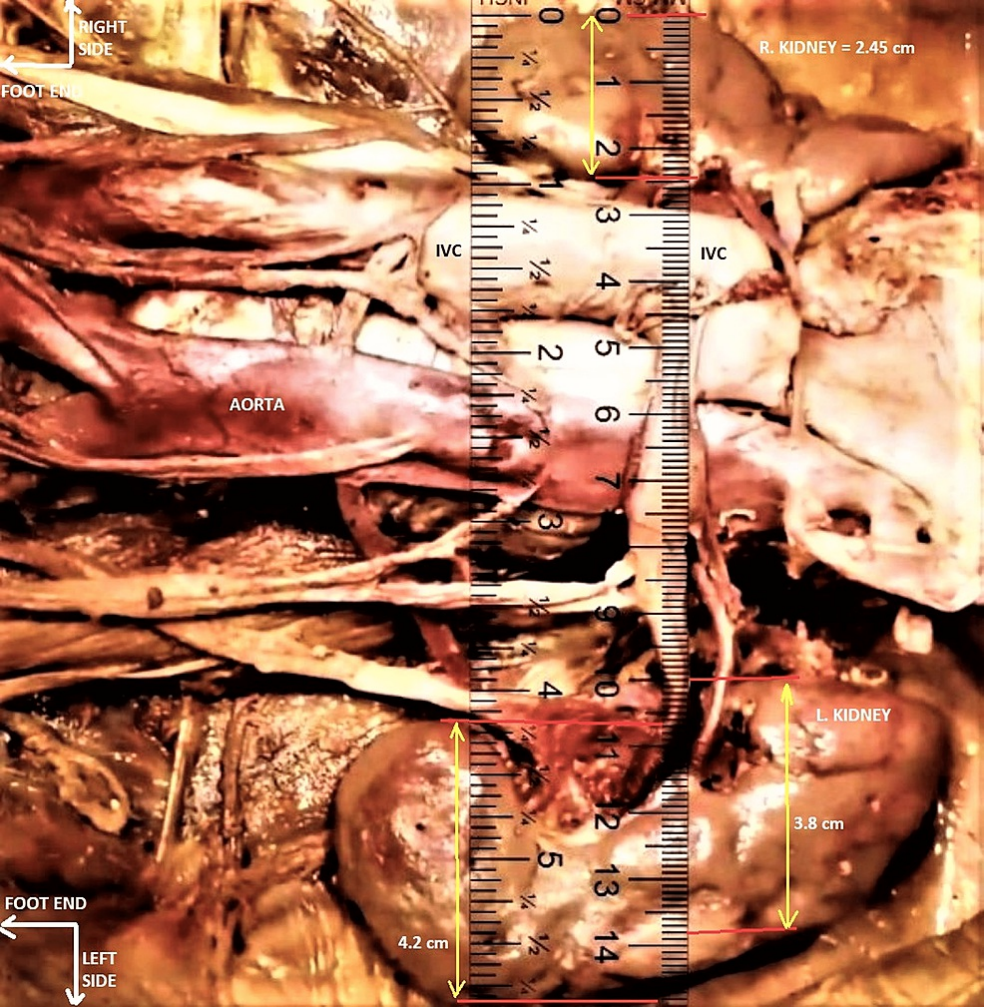
Measurement of the kidneys in situ using the online ruler (OR) app from Ginifab© The images of kidneys and the OR app itself were scaled to sizes to match the computer screen resolution (refer to text for details). The average dimensions of the right and left kidneys could be measured by this OR app, as shown in this sample image. The orientation of the subject is the same as in previous images. IVC - inferior vena cava

A proprietary online ruler (OR) app from Ginifab© (www.ginifab.com) that was freely available on the darknet web was used to record the dimensions of the kidneys in situ from the digital images. The app auto-calibrated itself to 100.7 pixels per inch (PPI) based on our screen resolution of 1536x864 pixels. High-resolution images of the dissected kidneys were uploaded on the app platform, and the digital OR was superimposed on the images. The digital controls on the app were adjusted, and both dissected kidneys were measured in situ in longitudinal and transverse axes. Right and left kidneys averaged 7.5 cm x 2.5 cm and 9.3 cm x 4.0 cm (length x width), respectively, as measured by the Ginifab© OR app (Figure 8).

**Figure 9:**
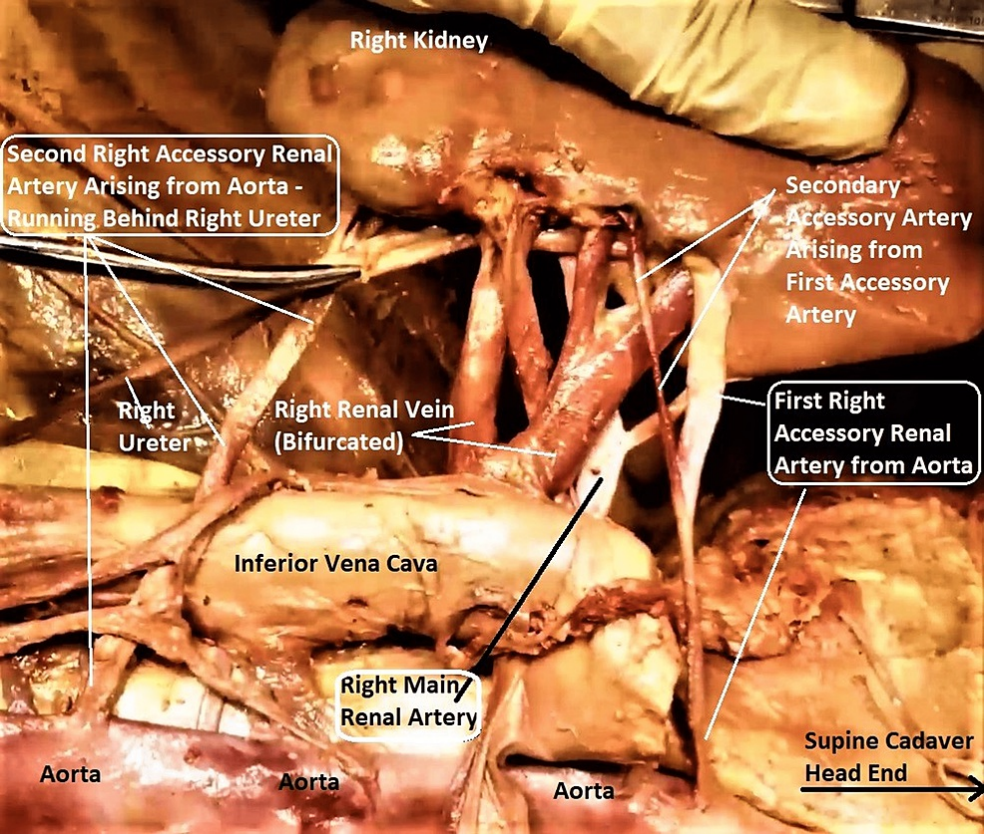
Dissected enlarged image of right renal vasculature in the subject Both accessory renal arteries (ARA) on the right side (labels enclosed in white rounded rectangular boxes) were long and narrow, arising independently from the aorta. The right lower ARA was passing behind the right ureter and entering the lower polar cortex separately from the main renal artery (MRA), which itself is normal. Note the "secondary accessory" branch arising separately from the right upper ARA (labeled with a white arrow) and entering the hilum. The orientation of the subject is the same as in previous images.

**Figure 10:**
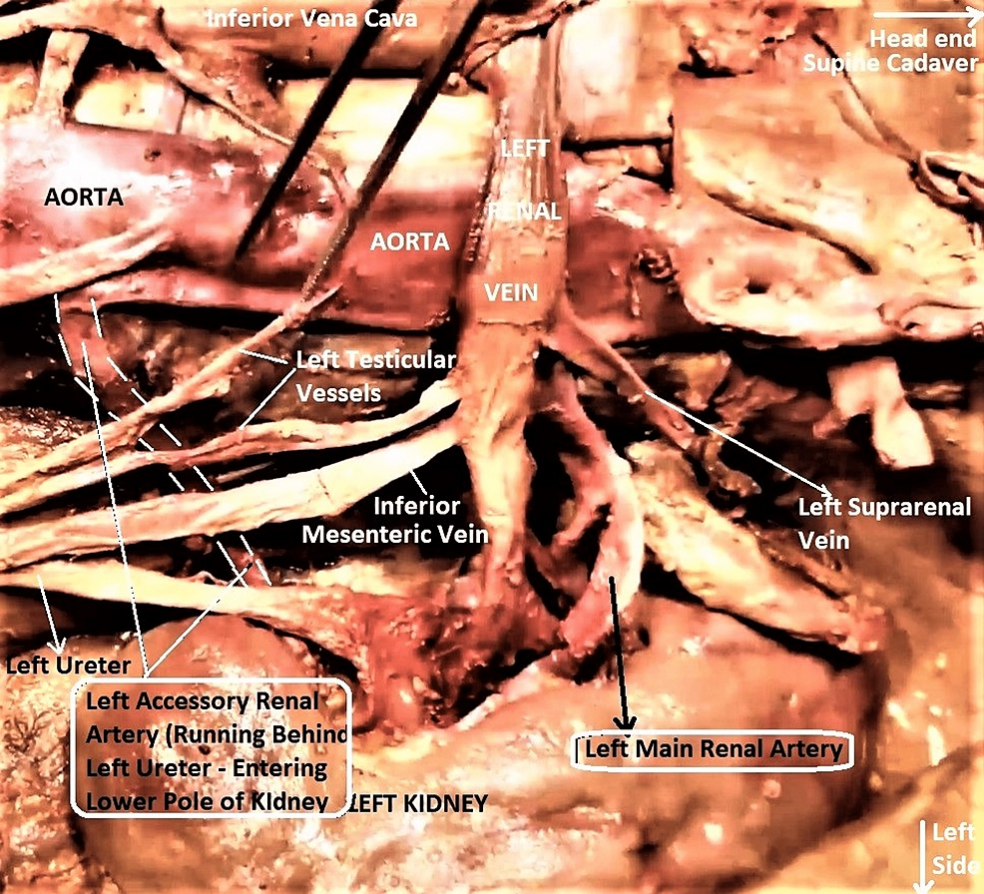
The dissected enlarged image of left renal vasculature in the subject The left accessory renal artery (ARA, outlined with double broken white lines) was thin and long, arising independently from the aorta, passing behind the left ureter and entering the left lower polar cortex separately from the main renal artery (MRA), which itself was normal. ARA and MRA labels are enclosed in white rounded rectangular boxes. The orientation of the subject is the same as in previous images.

The right kidney had two ARAs, arising directly from the aorta, above and below the right MRA, respectively. There was a "secondary accessory" artery arising from the right upper ARA itself. The right lower ARA was entering the right kidney separately through the lower polar cortex (Figure 9). The left kidney had one ARA arising from the aorta below the left MRA. This was also entering the left kidney independently through the lower polar cortex (Figure 10). All ARAs were longer and thinner than the corresponding MRAs on each side. A significant observation was that the lower ARAs on both sides were located posterior to the respective ureters (Figures 9-10).

## Discussion

Artificial urinary sphincter

The AUS explanted from our subject was a model belonging to AMS 800™ (American Medical Systems, Minnetonka, USA) that was introduced in 1983. The flat-backed cuff seen in this case report was introduced in 1987 [2,3]. Some authors have attributed the first AUS surgery to Frederick EB Foley [3]. In his 1947 paper, Foley (of "Foley's indwelling self-retaining catheter" fame) described a technique of creating a valve-like mechanism within the bladder neck by suturing a cuff of bladder mucosa to the surrounding detrusor musculature, which he termed as "artificial sphincter", though it was not a true sphincter [10]. The precursor of the current AUS was first devised by F. Brantley Scott and colleagues in 1972 [1]. Scott then went on to develop his own version of AUS in 1973 [11]. After the initial pioneering work, maximum developments in materials and techniques of AUS surgery occurred during the last two decades. Today AMS 800™ is universally considered as the gold standard in AUS implant technology [2,3,12]. The silicone elastomer material detected in our subject was derived from the National Aeronautics and Space Administration's (NASA's) high-fidelity, wear-resistant, non-irritant, non-toxic material research in the 1960s, which explained the lack of inflammation in our subject [2,3].

In vivo testing of the explanted AUS from our subject showed it was partially functioning, despite the rigors of embalming and the subsequent dissection process. In a living subject, pressing on the lower portion of the pump opens the inflow and outflow check valves inside. In this position, the deactivation valve poppet inside the pump is also open. This allows fluid to drain from the urethral cuff to the abdominal balloon, opening the urethra and allowing the subject to micturate. After three to five minutes, the deactivation poppet valve remains open, but the check valves get closed. This time the fluid flows slowly, regulated by the hydraulic resistor inside the pump, from the balloon back to the cuff, and the urethra closes, restoring urinary continence. Pressing the deactivation button on the upper part of the pump at any time closes the deactivation valve poppet, and fluid stops flowing in either direction [3,12]. While our subject's AUS was manually controlled, newer devices that are remotely controlled by neodymium magnet and Bluetooth are under testing [2].

AUS Cadaver Studies

An extensive literature search revealed seven AUS studies on human cadavers, ours being the eighth. The first report in 2012 compared perineal and trans-scrotal approaches for urethral cuff placement and concluded that though the latter was technically easier, the perineal approach offered better urethral size, the proximity of placement, and urethral coaptation as measured by urethral retrograde leak point pressure (RLPP) [13]. The second report in 2015 tested the feasibility of a newer ARTUS™ device (Affluent Medical, Paris, France) on cadavers [2]. The remaining five cadaveric reports, human and animal, tested the biomechanics, number, positioning, angulation, design, and efficiency of the urethral cuff [14-18]. Ours was the only cadaveric study that holistically checked AUS placement, functioning, possible complications, and probable indications of usage in the subject.

Placement of AUS

Considering the extent and density of fibrous tissue surrounding the AUS in our subject, the relative absence of urethral atrophy and urethral erosion, and the condition of the device itself, we estimated that it was implanted at least five years before the demise of the subject. Device survival goes down linearly, while the prevalence of complications resulting from the device increases proportionately five, ten, and fifteen years post-implant [2,3,12]. Our subject's device was in relatively good condition, without significant complications. The cuff was most probably implanted by the easier trans-scrotal approach in our subject, the pump itself being placed in the left sub-dartos pouch via the same approach. While some studies advocated the perineal approach [13], the scrotal approach appeared to be satisfactory in our subject. A significant observation was the location of the balloon anterior to the left lower RA muscle, just under the anterior rectus sheath in our subject. This would have necessitated a separate lower abdominal incision, as indicated by the density of fibrous tissue in this region in our subject. Urologists generally prefer the pre-vesical cave of Retzius or under the RA muscle or the preperitoneal space of Bogros for implanting the balloon. These sites can be accessed through the trans-scrotal incision. These balloon placement sites also generate a higher urethral RLPP during cuff inflation, providing better urinary continence because most patients are post-prostatectomy cases afflicted with stress urinary incontinence (SUI) [2,3,12,13]. It is our postulate, which we shall elaborate subsequently, that our subject perhaps did not require high urethral RLPP, which may have explained the balloon placement in his left lower anterior abdominal wall. His UI was physiologically driven rather than postoperative. Therefore, it was most probably moderate in severity.

Urinary Incontinence

The most common setting of SUI in adult males, which necessitates AUS implantation, is after trans-urethral resection of the prostate (TURP) for BPH, or after prostatectomy for cancer, with or without radiation [2,3,12]. Though, at the time of death, our subject was 78 years old, his bladder, trigone and bladder neck, and proximal urethra did not reveal any residual signs of BPH, cancer, surgery, or irradiation. The bladder interior and ureters did not show any evidence of back pressure in our subject. Other less common causes of male UI are neurological conditions (spinal cord lesions, Parkinson's disease), pelvic injuries, urinary tract infections (UTI), and medication usage. These latter conditions may cause urgency urinary incontinence (UUI) or overflow urinary incontinence (OUI). Our subject had no evidence of pelvic injuries or UTI. While we cannot speculate on the neurological aspects of our subject, knowledge of the subject's end-of-life ailments points to medications as the possible cause of UI. Antidepressants, antihistamines, and anti-hypertensives can cause UI as a side effect. Alpha-adrenergic antagonists may cause SUI. Agents with anticholinergic effects and anti-hypertensives acting on the central nervous system may cause urinary retention and OUI. Calcium-channel blockers, narcotics, and sedatives may also cause OUI and/or nocturnal enuresis. Beta-blockers and diuretics can cause bladder dysfunction, resulting in frequency and UUI [19,20]. It is our postulate that our subject was under some of these medications based on what was known about his cardiovascular and renal status.

Bilateral accessory renal arteries

ARA is an additional vessel that supplies blood to the kidney, in addition to the MRA. ARA may arise from the aorta, MRA, or any other source. Some have theorized that ARAs are remnants of sequential embryonic aortic branches during the developmental ascent of the kidneys. Our subject's distribution of three ARAs arising directly from the aorta on both sides conforms to this embryogenetic origin of ARAs. Single ARA is not uncommon, with a prevalence of 20-30% in various reports. Multiple and bilateral ARAs are rare, with a prevalence of 0.1-2% [4-6]. Our adult male subject had bilateral RA with bilateral ARAs, without any stenosis. The literature is sparse on this set of findings in male adults, and they are only individual case reports, attesting to their rarity [7,9].

ARAs may be associated with HTN, though there are dissenting opinions without sufficient hemodynamic evidence [7]. ARAs are generally long and thin, as they were in our subject. Hagen-Poiseuille's Law of Fluid Dynamics states that the rate of flow of fluid through a tube is directly proportional to the fourth power of its radius and inversely proportional to its length, as per the following equation;

$Q = \pi Pr^4 / 8\eta l$

Where, Q - flow; P - pressure difference; r - radius of tube; eta - viscosity of liquid; l - length of tube

Therefore, the relatively low intraluminal blood flow inside the ARAs may induce focal renal ischemia, which can initiate the renin-angiotensin-aldosterone (RAA) mechanism [7,8,21,22]. Given the bilateral atrophic kidneys associated with bilateral ARAs in our subject, it is possible he had RAA-induced HTN during his lifetime [23].

Bilateral renal atrophy 

The small size of both kidneys in our subject, flabby consistency, pitted, scarred surface, and absence of cortico-medullary differentiation on the longitudinal section all pointed to bilateral RA (Figures 4-6). Comparison with a normal kidney from another subject confirmed our diagnosis of RA (Figure 7). Adult RA is not to be confused with Ask-Upmark kidney. The latter refers to focal or segmental hypoplasia of one or both kidneys in hypertensive children, especially females, due to vesicoureteric reflux, pyelonephritis, or metanephric developmental arrest, with or without ARA or stenosis. Our subject did not qualify for this diagnosis [23].

Common causes of bilateral RA in adult males include chronic kidney diseases (CKD), which may be due to diabetes mellitus, HTN, or glomerulonephritis. Focal segmental glomerulosclerosis (FSGS) is the most common cause of nephrotic syndrome (NS) in African-American adult males. Our subject was of that ethnicity. NS can lead to bilateral RA. Long-term HTN can cause hypertensive nephrosclerosis (HNS), which can also cause RA. Any of these may have caused RA in our subject during his lifetime [24].

Clinicopathological reconciliation

**Figure 11:**
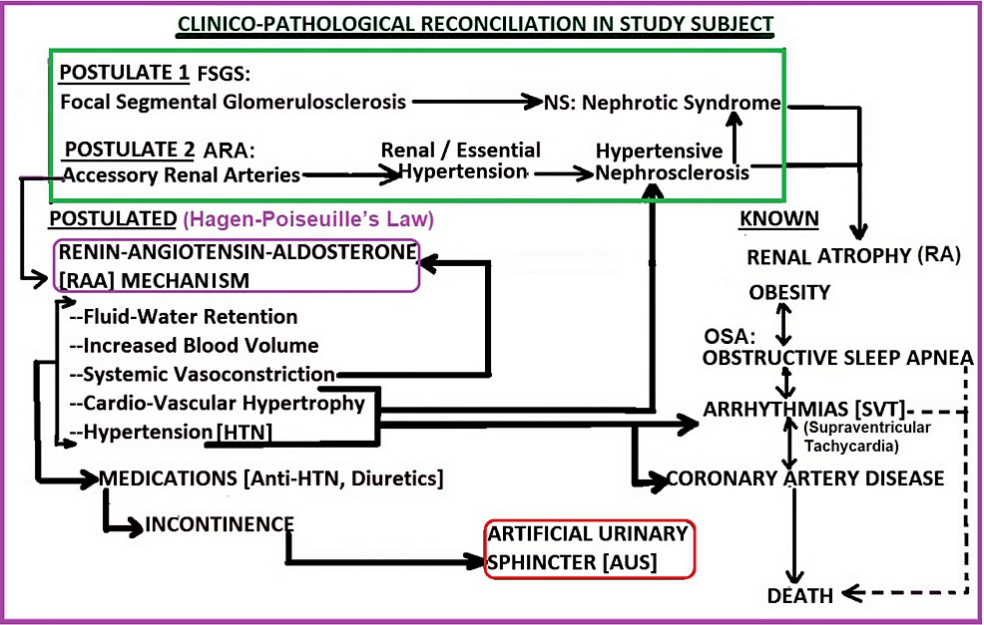
Schematic diagram highlighting the clinicopathological reconciliation in our subject This is an attempt to "close the loop" in the complex pathophysiological processes that may have been active in our subject during his lifetime by combining what was known about the subject, what was found at the time of dissection, and what was postulated from the dissection findings. This was the major focus of this report.

Our subject at the time of his demise was a 78-year-old obese African-American male. He had bilateral RA, three ARAs involving both sides and was fitted with an AUS for putative UI. The immediate cause of his death was CAD, with antecedent causes being SVT and severe OSA. We may postulate that our subject's RA may have been due to NS following FSGS or HNS [24]. The low blood flow in the long, thin ARAs may have initiated RAA mechanism by Hagen-Poiseuille's Law [7,8,22], leading to fluid retention and HTN, which would have worsened his renal status in a vicious cycle, necessitating the administration of diuretics, or anti-hypertensives [20,24]. These medications can precipitate UI as adverse effects [20], which necessitated AUS implantation in the subject during his lifetime. The normal appearance of the bladder and ureters leads us to speculate that obstructive uropathy was not a significant factor in our subject. Since the UI was not postoperative, an anterior abdominal wall balloon implant may have been sufficient in our subject [2,3,12,13]. Finally, the complex hemodynamic alterations resulting from his unique situation cumulatively compromised his cardiac status to the final end-of-life state (Figure 11).

## Conclusions

AUS surgery is a promising and exponentially increasing procedure worldwide, and a plethora of patient information leaflets are available on the subject. Given the circumstances of our subject, it behooves well for the physician to actively investigate any additional or underlying cardiovascular ailments, renal atrophy, abnormal renal vasculature, underlying causes of UI, and medication usage. These investigations may be but are not limited to, abdominal CT scan, renal CT angiogram, cardiac profile, and urodynamic studies. Armed with the extra information so gleaned, the physician would be able to make an informed decision about the type of AUS to implant, its functional lifespan and risks vis-à-vis patient's life expectancy, and any other intervention that may be required, concurrently or in tandem. 
